# Human exposure to *Anopheles farauti* bites in the Solomon Islands is not associated with IgG antibody response to the gSG6 salivary protein of *Anopheles gambiae*

**DOI:** 10.1186/s12936-019-2975-8

**Published:** 2019-10-01

**Authors:** Edgar J. M. Pollard, Catriona Patterson, Tanya L. Russell, Alan Apairamo, Jance Oscar, Bruno Arcà, Chris Drakeley, Thomas R. Burkot

**Affiliations:** 1 Australian Institute of Tropical Health & Medicine, James Cook University, Cairns, QLD 4870 Australia; 2 Department of Infection Biology, London School of Hygiene & Tropical Medicine, London, UK; 3 National Vector Borne Disease Control Program, Ministry of Health and Medical Services, Honiara, Solomon Islands; 4 Department of Public Health and Infectious Diseases, Division of Parasitology, Sapienza Università Di Roma, Rome, Italy

**Keywords:** gSG6, Human biting rate, *Anopheles farauti*, Saliva antigens

## Abstract

**Background:**

Mosquito saliva elicits immune responses in humans following mosquito blood feeding. Detection of human antibodies recognizing the *Anopheles gambiae* salivary gland protein 6 (gSG6) or the gSG6-P1 peptide in residents of Africa, South America and Southeast Asia suggested the potential for these antibodies to serve as a universal marker to estimate human biting rates. Validating the utility of this approach requires concurrent comparisons of anopheline biting rates with antibodies to the gSG6 protein to determine the sensitivity and specificity of the assay for monitoring changes in vector populations. This study investigated whether seroprevalence of anti-gSG6 antibodies in humans reflected the relative exposure to *Anopheles farauti* bites in the Solomon Islands as estimated from sympatric human landing catches.

**Methods:**

Human biting rates by *An. farauti* were estimated by landing catches at 10 sampling sites in each of 4 villages during the wet and dry seasons. Human serum samples from these same villages were also collected during the wet and dry seasons and analysed for antibody recognition of the gSG6 antigen by the Luminex xMAP^©^ platform. Antibody titres and prevalence were compared to HLCs at the sampling sites nearest to participants’ residences for utility of anti-gSG6 antibodies to estimate human exposure to anopheline bites.

**Results:**

In this study in the Solomon Islands only 11% of people had very high anti-gSG6 antibody titres, while other individuals did not recognize gSG6 despite nightly exposures of up to 190 bites by *An. farauti*. Despite clear spatial differences in the human biting rates within and among villages, associations between anti-gSG6 antibody titres and biting rates were not found.

**Conclusions:**

Few studies to date have concurrently measured anopheline biting rates and the prevalence of human antibodies to gSG6. The lack of association between anti-gSG6 antibody titres and concurrently measured human biting rates suggests that the assay for human anti-gSG6 antibodies lacks sufficient sensitivity to be a biomarker of *An. farauti* exposure at an epidemiologically relevant scale. These findings imply that an improvement in the sensitivity of serology to monitor changes in anopheline biting exposure may require the use of saliva antigens from local anophelines, and this may be especially true for species more distantly related to the African malaria vector *An. gambiae*.

## Background

As malaria transmission diminishes, endemic areas become stratified with foci of residual transmission appearing, based on receptivity, vulnerability and access and use of malaria prevention strategies [e.g., long-lasting insecticide-treated nets (LLINs) and indoor residual spraying (IRS)] [1, 2]. Defining foci and receptivity becomes increasingly challenging in areas with small vector populations. The classic entomological measure of transmission intensity, the entomological inoculation rate (EIR), requires estimating two parameters (the biting rate and the sporozoite rate), both of which can vary widely and rapidly in time and space. Hence, the EIR best estimates transmission intensity in high malaria transmission settings with high mosquito biting populations [3, 4]. Due to the lack of precision in estimating sporozoite rates in low-transmission settings, vector biting rates remain the best entomological proxy to estimate receptivity despite the logistical challenges associated with spatial–temporal heterogeneity in biting rates [5].

Recently, human antibodies recognizing the *Anopheles gambiae* salivary protein gSG6 [6] or the gSG6-P1 peptide [7] were shown to be associated with recent exposure to anopheline bites in tropical Africa [8, 9]. A similar, although weaker, association was later documented in a number of geographic areas, including South America and Southeast Asia where other anopheline species are responsible for malaria transmission [10, 11]. In the South Pacific nation of Vanuatu, seroprevalence to gSG6 was correlated to reactivity to *Plasmodium falciparum* and *Plasmodium vivax* antigens, which was hypothesized to be related to exposure to the bites of *Anopheles farauti*, but biting rates were not estimated [12]. These observations suggested that anti-gSG6 antibody levels or seroprevalence might serve as an entomological proxy to estimate anopheline biting intensity by reflecting recent exposure to anopheline bites [8]. This approach has advantages compared to estimating biting rates by mosquito counts, as antibody prevalence would be less affected by short-term fluctuations in transmission intensity relative to estimates of the number of biting mosquitoes and would therefore be more cost effective and potentially more precise.

In the Western Province of the Solomon Islands, malaria transmission is heterogeneous with a wide range in *An. farauti* biting rates documented [5]. The potential of anti-gSG6 antibodies to be a biomarker of human exposure to *An. farauti* bites in the Western Province, Solomon Islands was investigated*.*

## Methods

**Fig. 1:**
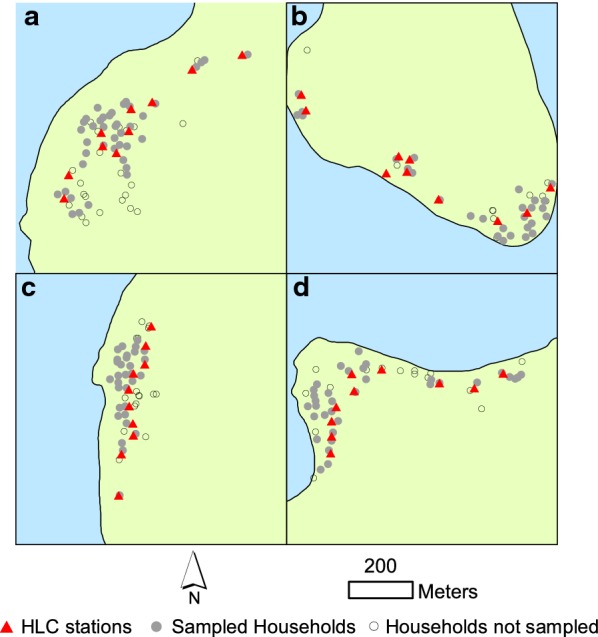
Proximity of stations where *Anopheles farauti* biting rates were estimated by human landing catch to households of individuals providing serum are shown for the villages of New Mala (**a**), Jack Harbour (**b**), Tuguivili (**c**) and Saeragi (**d**)

Mosquito and human blood surveys took place during the dry and wet seasons in the villages of Jack Harbour, Tuguivili, Saeragi, and New Mala in Western Province, Solomon Islands [5, 13]. Human biting rates were estimated twice in the dry season (May and August 2016) and twice in the wet season (November 2016 and February 2017). Because *An. farauti* biting densities are heterogeneous within and among villages, biting rates were estimated by human landing catches (HLC) at 10 locations (stations) within each village from 18:00 to 00:00 h. During this period, 93% of bites by *An. farauti* occurs [14]. Hence, this collection period closely approximates the number of bites by *An. farauti* nightly and will be referred to hereafter as the nightly biting rate. During each village vector survey, sampling was conducted for four nights at each of 10 sampling locations (Fig. 1). The mosquitoes were identified morphologically in the field [15] and then a sub-sample was further identified by PCR amplification of the internal transcribed spacer region 2 of the ribosomal DNA for molecular confirmation of species [16].

Human blood surveys were conducted in September 2016 and March 2017. After explaining the purpose of the survey, including potential risks, blood samples were taken from all individuals providing written consent (consent for individuals < 18 years was provided by a parent or guardian). Demographic data (name, age, gender, house location, travel history, anti-malaria, and bed-net use histories) were recorded prior to collection of blood samples by finger prick. Samples were allowed to clot before centrifugation to separate serum. Serum was removed and transferred into 2-mL micro-vials labelled with a unique identifying number. Sera was initially stored at 4 °C, and within 4 days moved to a central location and frozen before shipping on dry ice with subsequent storage at ‒ 80 °C.

Anti-gSG6 antibody titres were measured using an IgG detection quantitative suspension bead array on the Luminex xMAP^©^ platform (Luminex Corp, Austin, TX, USA) alongside a panel of *P. falciparum* (PfAMA1, PfMSP119 and GLURP2)- and *P. vivax* (PvAMA1 and PvMSP1-19)-derived recombinant antigens. The Luminex assay for gSG6 antibodies was optimized by testing a range of gSG6 concentrations coupled to Luminex microspheres against 4 control pools for reactivity, specificity and optimal coupling concentration. Assay conditions were validated against serum samples from Burkina Faso with high and low mosquito bite exposure along with British negative controls and malaria-positive controls. Serum dilutions of 1:200 were used in subsequent assays to minimize background cross-reactivity with an optimal concentration of 18.28 μg/mL of gSG6-P1 coupled to microsphere beads.

Diluted samples (1:200) were co-incubated with gSG6-coated microspheres, and after washing, a detection antibody was added followed by a final wash. The median fluorescent intensity (MFI) emitted by a reporter molecule on the detection antibody was measured using a Luminex MAGPIX^©^ analyzer. All antigens underwent pre-assay optimization to identify a coating concentration that captured a dynamic range of responses [17]. Control sera consisted of hyperimmune serum samples to *P. falciparum* and *P. vivax*, and British malaria-naïve individuals.

Positives, negatives and blanks were present on each plate and compared to each other and pre-existing data to test for variability and accuracy. The positive control panel consisted of 10/198 (WHO *P. falciparum* positive reference standard curve), two duplicate wells of CP3 (a *P. falciparum* positive pool sourced from hyperimmune Tanzanian individuals) and two duplicate wells of 72/96 (a *P. vivax* positive control) at 1:200 dilutions. Negative controls consisted of 4 malaria-naive samples per plate (from a panel of 40 samples provided by Public Health England). Two wells of each plate contained only dilution buffer to test for background reactivity. A seropositive sample was defined as having an MFI value greater than the mean value of the negative samples plus three standard deviations.

The dataset was analysed by pairing individual gSG6 antibody MFI values with biting rate estimations based on the locations of individuals’ home residences as 82% of exposure to biting *An. farauti* occurs either in the home or the adjacent peri-domestic area (Pollard et al*.* submitted). Each participant providing a blood sample was assigned an anopheline biting rate based on the mean nightly HLC of the nearest HLC station to that individual’s house. Participants living more than 100 m from an HLC station were excluded in the analyses for associations between biting rates and antibodies to gSG6. The relationship between biting exposure (estimated from HLC) and antibody titres was analysed using Spearman’s rank correlation. The differences in biting rates between villages and seasons were analysed using generalized linear models (GLM). The difference between the gSG6 MFI values for each village was analysed using a GLM with the British control group as the reference. For the residents that provided serum during both the dry and wet season, a paired dataset was constructed and the change in the gSG6 antibody MFI values over time was compared with a Wilcoxon signed ranked test. Statistical analysis was conducted using R statistical software (ver. 3.1.2).

## Results

A total of 10,110 female anophelines were sampled during 660 HLC collections at 10 stations in each of 4 villages (Fig. 1). Species identification was confirmed by PCR in a sample of 630; 95% of the confirmed species identifications were *An. farauti* (601 of 630), 3.5% were *Anopheles hinesorum* (22 of 630) and 1% were *Anopheles lungae* (7 of 630). The mean biting rate of *An. farauti* varied significantly by village (Table 1; *β* = 24.740, se = 1.1216, p < 0.0001) and season (*β* = 6.4063, se = 1.1917, p < 0.0001). The average nightly biting rate of anophelines during the dry season across all 4 villages was 6.5 compared to an average of 19.0 during the wet season. Highest biting rates were in Jack Harbour, where only *An. farauti* was found. Mean biting rates among 10 sampling stations in Jack Harbour ranged to 190 (Table 1).

**Table 1:** The mean and range in *Anopheles farauti* human landing rates from 10 sampling sites within each village during 4 surveys

Season	Date	Village [mean (range)]			
		Jack Harbour	New Mala	Saeragi	Tuguivili
Dry	May 2016	44.0 (7–120)	2.7 (0–14)	0.2 (0–2)	1.8 (0–8)
	Aug 2016	1.1 (0–6)	1.8 (0–10)	0.1 (0–2)	0.3 (0–4)
Wet	Nov 2016	67.4 (0–367)	0.2 (0–3)	0 (0–0)	12.9 (0–73)
	Feb 2017	64.6 (2–279)	0.6 (0–4)	0 (0–0)	6.7 (0–26)
	Mean	47.7	1.2	0.1	5.7

A total of 791 serum samples were collected from residents of the 4 villages. From each village, Jack Harbour, New Mala, Saeragi, and Tuguivili, the number of samples collected in the dry season (September 2016) were 74, 117, 110, and 85, respectively; and in the wet season (March 2017) were 69, 137, 83, and 116, respectively. Overall, a total of 210 samples were paired being collected from the same 105 residents during the dry season and again in the wet season.

**Fig. 2:**
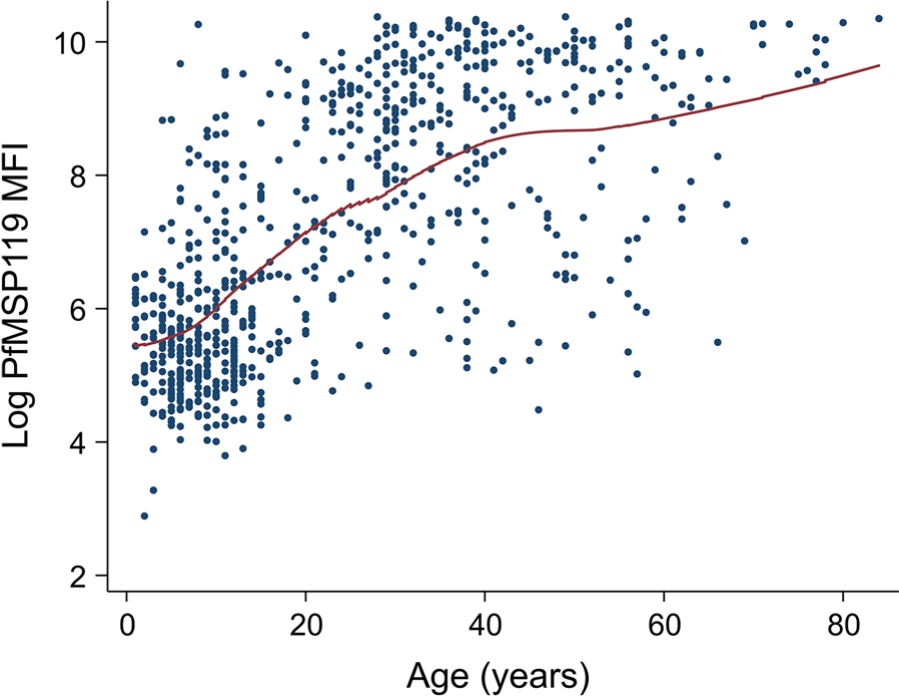
Age-specific patterns of long-term *Plasmodium falciparum* exposure of residents in Western Province of the Solomon Islands represented by a scatter plot of log-transformed antibody PfMSP1-19 median fluorescence index (MFI) by age with loess regression line (red line)

**Fig. 3:**
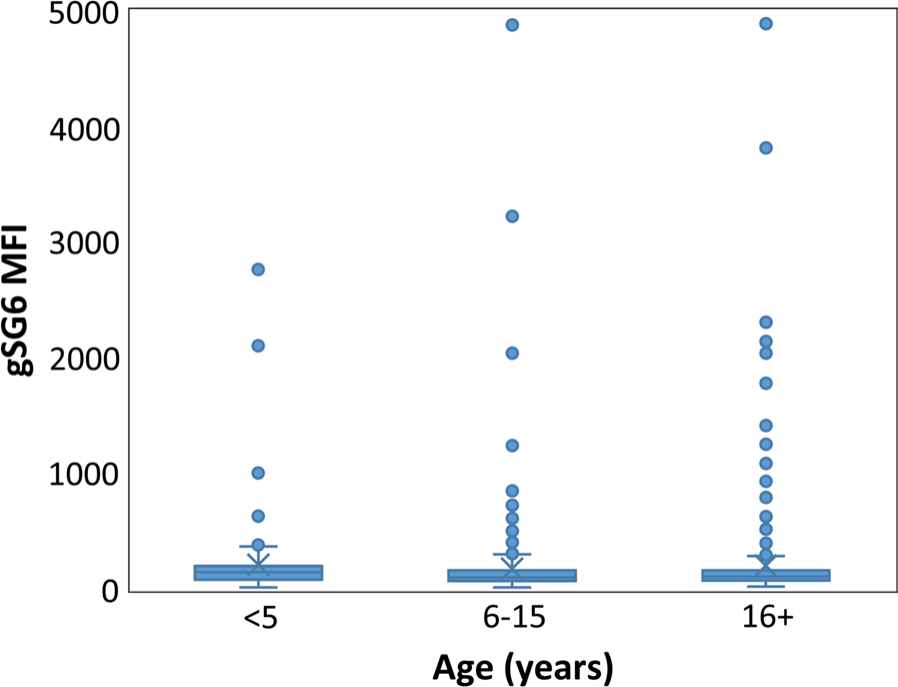
Age-specific patterns of gSG6 MFI recognition for serum of residents of Western Province in the Solomon Islands (sample sizes: < 5 years, n = 98; 6–15 years, n = 260; > 16 years, n = 424). Differences in mean gSG6 MFI reactivity by age were not significantly different

Typical age-specific patterns of long-term exposure markers, PfAMA1, PfMSP1-19, GLURP2, PvAMA1 and PvMSP1-19 were observed for the serum samples from the Solomon Islands (results for PfMSP1-19 shown in Fig. 2), confirming the assay performed as expected for the parasite antigens. When gSG6 MFI values were analysed by age categories (< 5, 6–15 and > 16 years), no significant association was seen (Fig. 3; β = ‒ 18.35, se = 22.32, *p* = 0.411). The gSG6 responses of the negative control panel of 40 malaria-naïve samples fell within a range of the mean plus three standard deviations (MFI value of 253).

Estimates of mean *An. farauti* bite exposures of individuals were based on HLCs at the nearest mosquito collection station to an individual’s house (Fig. 1). Participants living within 100 m of the nearest mosquito collection station (n = 733 samples) were included in the analyses for associations between biting rates and antibodies to gSG6. The mean distance from houses to closest HLC station for participants was 29 m (mean distances by village were 23 m, 36 m, 33 m, and 25 m in Jack Harbour, New Mala, Saeragi and Tuguivili, respectively).

**Fig. 4:**
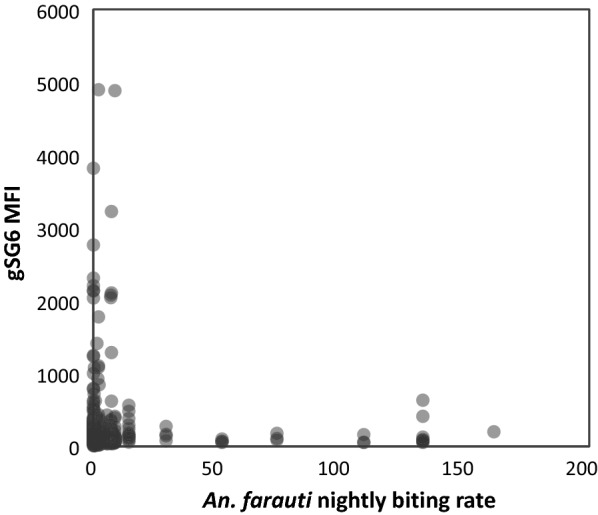
The relationship between the intensity of anti-gSG6 antibody responses and exposure to the mean number of *Anopheles farauti* bites per person per half-night estimated from the nearest collection station to participants homes during the month preceding blood surveys

Sera from 11% of 791 samples were classified as seropositive to gSG6. A significant relationship between the prevalence of anti-gSG6 antibody MFI and intensity of exposure to *An. farauti* bites in the month preceding blood surveys was not found (Fig. 4) (ρ = 0.0276, p = 0.4). In fact, residents with high nightly *An. farauti* exposure (> 10 bites) did not generate high levels of antibody titres: the highest gSG6 MFI value observed was 634 for individuals exposed to high biting rates, while residents exposed to more moderate biting rates (< 10 bites/night) had gSG6 MFI values ranging up to 4897. Analyses comparing the prevalence of anti-gSG6 antibody MFI and intensity of exposure to *An. farauti* bites in both the wet and dry seasons as the mean of HLCs in surveys one and four months preceding each blood survey also did not reveal any significant relationships (ρ = ‒ 0.0702, p = 0.05).

**Fig. 5:**
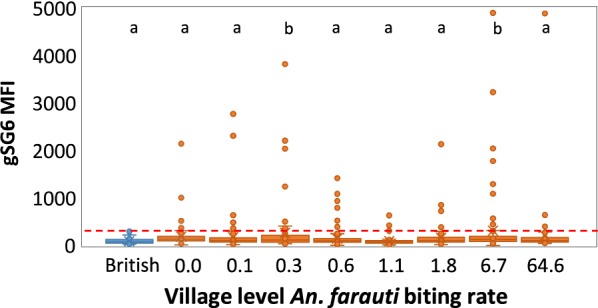
The relationship between the intensity of anti-gSG6 MFI antibody responses of individuals by estimated *An. farauti* biting rates from the biting surveys a month before the blood survey. Seropositives were defined as sera generating an MFI greater than the mean plus three standard deviations of the MFI values from 40 malaria naïve British (shown as blue). Seropositive cut-off line is shown as a red dashed line. Significantly different mean MFI values (at the 002 level) of participants of villages are identified by different letters above the village specific MFI values. Significantly elevated MFI values relative to British sera were found in two village surveys when biting rates were low (0.3 bites/night) and high (6.7 bites/night)

Differences in the population level antibody titres for each village were analysed both in the dry and wet seasons using the British population as a reference group (Fig. 5). There were only two Solomon Island populations (Tuguivili village during both the dry and wet seasons when nightly biting rates were 0.3 and 6.7) that were statistically different from the British control serum samples (*p* = 0.022 and *p* = 0.002). In the other villages, MFI values were not significantly different from the British controls, including Jack Harbour village with an almost tenfold greater nightly number of biting *An. farauti* than Tuguivili village (Fig. 5).

Overall, significant differences in the mean MFI to gSG6 of all serum samples between the dry and wet seasons were not found. However, when paired sera of individuals (n = 105) from the dry and wet seasons were compared, there was a slight but significant increase in MFI values to gSG6 from the dry (mean = 189) to the wet season (mean = 202.9) (Wilcoxon signed ranked test: V = 1982.5, p = 0.01542). The MFI values to gSG6 of residents of Jack Harbour showed a significant increase between the dry and wet seasons, increasing from a mean of 74.08 to 125.8 (Wilcoxon signed ranked test: V = 17, p = 0.0001) when the mean nightly *An. farauti* biting rates in the dry and wet seasons increased from 22.5 to 66.5 (based on the mean of two vector surveys for HLC preceding the blood survey).

## Discussion

An association between human anti-gSG6 antibody titres (expressed as MFI values) and exposure to *An. farauti* bites as determined by HLCs was not observed in this study. While some individual serum samples strongly recognized gSG6, the reactivity of most serum samples from the malaria-endemic Solomon Islands were not significantly greater than the British negative controls. Failure to find significant recognition of anti-gSG6 antibodies in the majority of residents tested could be due to epidemiological factors (e.g., inadequate exposure to *An. farauti* bites, or variations in the attractiveness of individuals to *An. farauti* not captured by the estimates of human biting rates) or immunological effects (e.g., limited understanding of the kinetics of the anti-gSG6 response in humans, inadequate assay sensitivity or insufficient similarity of saliva antigens of *An. farauti* to the gSG6 antigen of *An. gambiae*). Previous studies indicated that antibody levels and/or seroprevalence to vector salivary antigens can reliably estimate malaria transmission in a number of countries, especially in tropical Africa (i.e., seroprevalence to gSG6 or gSG6-P1 and malaria antigen markers are significantly associated). However, validating the IgG response to gSG6 as a reliable marker of mosquito exposure requires comparing human antibody reactivity to gSG6-P1 or to gSG6 with estimates of mosquito biting rates on the same individuals or by comparing the reactivity of populations in defined areas with estimates of biting rates in the same areas, as was done in Senegal and Cambodia using single estimates of biting rates for each census district or village, respectively [9, 18]. In this study in the Solomon Islands, biting rates within villages were determined at 10 locations per village by HLCs to estimate the exposure of residents of nearest houses to biting mosquitoes.

In Cambodia, despite a ninefold range in the size of *Anopheles dirus* populations between two villages, little difference was seen in the corresponding antibody recognition of gSG6 [18]. In this study, a greater than 100-fold range in exposures to *An. farauti* bites was documented amongst 4 villages without finding an association with the titre or prevalence of antibodies to the gSG6 antigen.

Previous work in Senegal found an association between antibody prevalence to whole *An. gambiae* saliva or to the gSG6-P1 peptide and *An. gambiae* nightly biting rates up to 124 [9, 19]. This biting intensity is comparable to the nightly exposure of people to *An. farauti* bites in the high exposure village in this study (191 bites/person). While a minority of individuals had a wide range in seropositive antibody titres recognizing gSG6 in the Solomon Islands, most individuals (89%) were seronegative. Amongst the 11% of individuals who were seropositive, significant associations with *An. farauti* bite exposure were not seen. In Vanuatu where *An. farauti* is also the primary vector, a decrease in seroprevalence to gSG6 with malaria transmission was correlated to reactivity to *P. falciparum* and *P. vivax* antigens [12]. A significant difference between the study in Vanuatu and this study in the Solomon Islands was that the *An. farauti* biting rates in Vanuatu were not estimated entomologically whereas the biting rates in the Solomon Islands were estimated at a fine scale enabling specific estimates of the exposure of individuals to biting *An. farauti* with the intensity of antibody recognition of gSG6 by those same individuals.

These results could be explained in part by a lack of assay sensitivity, as hypothesized by the studies in Cambodia [18] and Vanuatu [12]. Previous studies employed gSG6-P1 or gSG6 as antigens with sera diluted from 1:20 to 1:200. The studies in Cambodia and Vanuatu measured responses in an ELISA to 5 µg/mL gSG6 at a serum dilution of 1:200. This study also measured antibody responses of serum diluted 1:200 to gSG6, but in a Luminex platform. While anti-gSG6 antibody prevalence may be lower due to the serum concentration used in the assays (1:200), any relationship between exposure to high levels of anopheline bites and corresponding highly reactive sera to gSG6 should still have been evident. Furthermore, a typical age-related increase in antibodies to specific malaria antigens was observed in this study in the Solomon Islands suggesting the assay performed as expected. A lack of age-related antibody response for gSG6 was observed in Burkina Faso but was hypothesized to be immune tolerance generated after intense and prolonged exposure to bites of Afrotropical malaria vectors [20, 21].

Another plausible explanation for the lack of association between anti-gSG6 antibodies and *An. dirus* biting rates in Cambodia [18] and in this study with *An. farauti* may be the limited sequence homology between *An. gambiae* and both *An. dirus* and *An. farauti* SG6 proteins. The *An. gambiae* gSG6 (used as antigen in all reported studies) shares only 54 and 52% identity with *An dirus* and *An. farauti* SG6 proteins, the primary malaria vectors in Cambodia and the Solomon Islands, respectively [22]. The limited similarity (70%) to *An. farauti* gSG6 was hypothesized as likely responsible for a low assay sensitivity in the Vanuatu study [12].

In the study in the Americas, antibody recognition of the gSG6-P1 antigen was reported as significantly correlated with malaria infection status and mosquito bite exposure history of Columbians and Chilians [10]. In that study, residents of Columbia may have been exposed to the bites of the main malaria vectors, *Anopheles albimanus*,* Anopheles darlingi* and *Anopheles punctimacula*; Chilean soldiers stationed in Haiti would have been potentially exposed to *An. albimanus*, the only malaria vector in Haiti. However, the SG6 antigen is absent in both *An. albimanus* and *An. darlingi* as well as in all other species belonging to the *Nyssorhynchus* sub-genus analysed [22, 23], suggesting that the antibodies to gSG6 in Columbians may represent exposure to *An*. *punctimacula* (a member of the *Anopheles* sub-genus) or minor vectors. For the Chilean soldiers, previous exposure to anopheline bites in Chile, including *Anopheles pseudopunctipennis* (also belonging to the *Anopheles* sub-genus), may have generated the antibody recognition of gSG6 [24]*.* While *Anopheles atacamensis* and *Anopheles pictipennis* are also endemic to Chile [25], these species are in the same sub-genus (*Nyssorhynchus*) as *An. albimanus* or *An. darlingi* which lack the SG6 protein coding gene [22, 23].

The use of antibodies against anopheline salivary proteins as markers of human exposure to malaria vectors has multiple advantages over longitudinal collections of anophelines by HLCs; in fact, serological analyses are both faster (requiring a single cross-sectional survey to estimate recent exposure to biting mosquitoes) and less expensive while minimizing exposure of survey teams to anophelines bites.

In the Southwest Pacific where *An. farauti* is the dominant vector, a number of challenges to using and interpreting antibody recognition of the gSG6 antigen as a proxy for measuring biting exposure were identified that limits the potential of this assay for programme or research applications. Firstly, the overall low reactivity of most sera of Solomon Islands residents to gSG6 will require large numbers of serum samples to detect significant changes in biting intensity. Consequently, the assay will not be applicable for monitoring small-scale heterogeneities in biting rates (a feature of low transmission scenarios in general and in the Southwest Pacific in particular [5, 26]. Secondly, finding highly reactive serum in villages with very low *An. farauti* biting rates and, conversely, unreactive serum in village residents with very high biting rates is perplexing. This will require well-characterized studies to compare serum reactivity to saliva antigen with concurrent biting rates to understand the relationship between bite exposure and the development (and loss) of antibodies to saliva antigens to allow epidemiologically relevant interpretations of changes in the prevalence and intensity of antibody recognition of saliva antigens.

## Conclusion

Despite the fact that significant levels of anti-gSG6 antibodies were not found in individuals exposed to significant numbers of *An. farauti* bites in this study, the potential utility of human antibodies as markers of biting exposure shows great promise. However, as pointed out by the results reported here, the use of anopheline salivary antigens as a proxy for estimating human exposure to bites of malaria vectors may require the use of salivary antigens from local mosquito species and validation by correlation of antibody reactivity with concurrent entomological measurements.

## Data Availability

The datasets supporting the conclusions of this article are available in the JCU Tropical Data Hub repository: https://doi.org/10.25903/5d4a47cbf1df2.

## Abbreviations

HBR: human biting rate
HLC: human landing catch
EIR: entomological inoculation rate
MFI: median fluorescence index
b/p/hn: bites per person per half night
